# A TIGIT-based chimeric co-stimulatory switch receptor improves T-cell anti-tumor function

**DOI:** 10.1186/s40425-019-0721-y

**Published:** 2019-09-09

**Authors:** Shiran Hoogi, Vasyl Eisenberg, Shimrit Mayer, Astar Shamul, Tilda Barliya, Cyrille J. Cohen

**Affiliations:** The Laboratory of Tumor Immunology and Immunotherapy, The Mina and Everard Goodman Faculty of Life Sciences, Bar-Ilan University, 52900-02 Ramat Gan, Israel

**Keywords:** TIGIT, T-cell engineering, Tumor immunotherapy, Costimulation, Chimeric receptors

## Abstract

**Background:**

Tumors can employ different mechanisms to evade immune surveillance and function. Overexpression of co-inhibitory ligands that bind to checkpoint molecules on the surface of T-cells can greatly impair the function of latter. TIGIT (T cell immunoreceptor with Ig and ITIM domains) is such a co-inhibitory receptor expressed by T and NK cells which, upon binding to its ligand (e.g., CD155), can diminish cytokine production and effector function. Additionally, the absence of positive co-stimulation at the tumor site can further dampen T-cell response.

**Methods:**

As T-cell genetic engineering has become clinically-relevant in the recent years, we devised herein a strategy aimed at enhancing T-cell anti-tumor function by diverting T-cell coinhibitory signals into positive ones using a chimeric costimulatory switch receptor (CSR) composed of the TIGIT exodomain fused to the signaling domain of CD28.

**Results:**

After selecting an optimized TIGIT-28 CSR, we co-transduced it along with tumor-specific TCR or CAR into human T-cells. TIGIT-28-equipped T-cells exhibited enhanced cytokine secretion and upregulation of activation markers upon co-culture with tumor cells. TIGIT-28 enhancing capability was also demonstrated in an original in vitro model of T-cell of hypofunction induction upon repetitive antigen exposure. Finally, we tested the function of this molecule in the context of a xenograft model of established human melanoma tumors and showed that TIGIT-28-engineered human T-cells demonstrated superior anti-tumor function.

**Conclusion:**

Overall, we propose that TIGIT-based CSR can substantially enhance T-cell function and thus contribute to the improvement of engineered T cell-based immunotherapy.

**Electronic supplementary material:**

The online version of this article (10.1186/s40425-019-0721-y) contains supplementary material, which is available to authorized users.

## Background

T-cell activation and function are dependent on multiple signals. First and foremost, a specificity signal mediated by the TCR (T-cell receptor) upon recognition of a specific antigenic peptide presented by MHC molecules is needed to activate the cell. In addition, co-stimulatory/inhibitory molecules can supply a second signal that can impact on T-cell function, proliferation and response. Amongst the different receptors participating in this second signal, CD28, ICOS, 4-1BB etc. are considered co-stimulatory and CTLA4, PD1, LAG3, Tim-3, and TIGIT enforce an inhibitory phenotype [1, 2]. The latter, TIGIT (T cell immunoreceptor with Ig and ITIM domains), is a checkpoint molecule that belongs to the poliovirus receptor (PVR)/nectin family and it was identified by Yu and colleagues [3]. TIGIT is expressed by lymphocytes, mainly by NK cells, CD4^+^, CD8^+^ and regulatory T cells (T_reg_). It is crucial for balancing T cell activation and for protection from autoimmunity [4–7].

Similarly to the antagonistic relationship of CTLA-4/CD28 with their ligands, TIGIT competes with a “positive” (stimulatory) receptor CD226 (also known as DNAM1). Both can bind to either of the two following ligands, CD155 and CD112, though TIGIT does so with a higher affinity [8–10]. It is important to mention that TIGIT also binds to CD155 with higher affinity than CD112 [4, 11]. TIGIT expression on naïve T cells is usually low, though it is upregulated following activation [10], and particularly on exhausted T cells in the tumor microenvironment (TME) [12]. TIGIT was shown to inhibit T cell proliferation and activation upon binding to CD155 [13, 14]. Similarly to other immune checkpoint ligands, TIGIT ligands are often overexpressed in cancer cells [15–18] while TIGIT is significantly upregulated in chronically stimulated or exhausted tumor-infiltrating T cells [14, 19, 20]. TIGIT activation can reduce NK cell cytotoxicity [21] and CTL proliferation and cytokine production via SHIP1-mediated mechanisms causing downstream inhibition of NF-kB, PI3K and MAPK pathways, and thereby diminishing the effectiveness of the cellular immune response [10, 13, 22, 23]. Moreover, high TIGIT expression on CD8^+^ T cells is associated with diverse malignancies include gastric cancer [12] and refractory hematological cancer and their relapse [14, 19, 20]. Thus, TIGIT represents an attractive target for immunotherapeutic intervention.

In the past decade, a tremendous progress was achieved in the treatment of cancer due to the development of immunotherapeutic approaches that include the use of checkpoint inhibitors, personalized cancer vaccines and the adoptive cell transfer (ACT) of tumor specific lymphocytes (either tumor infiltrating or genetically engineered T-cells) [24]. T-cell engineering was primarily designed to endow T cells with novel specificities, and this can be achieved by expressing either a T cell receptor (TCR) or a chimeric antigen receptor (CAR) consisting of a targeting moiety (e.g., scFv) fused to an activation domain (that incorporate usually a co-stimulation portion and the CD3ζ intracellular domain).

An important difference between native TCR and CAR is the inclusion of co-stimulatory domain(s) in the latter. To recruit co-stimulation in the context of TCRs, it is possible to either transduced them with CD28 or 4-1BB [25, 26], provided their respective ligands are expressed by the target cells. Another approach is based on the use of chimeric costimulatory switch receptor (CSR) based on the exodomain of coinhibitory receptors and the endodomain of costimulatory ones [27]. We and others demonstrated that CSRs based on PD1 can enhance T-cell function in the presence of inhibitory ligands expressed by tumors cells [28, 29]. Whereas therapeutic approaches that target immune checkpoint receptors such as CTLA4 and PD-1 have demonstrated unprecedented results in cancer patients, not all of them will eventually benefit from these treatments [10]. Thus, it is desirable to assess the impact of targeting additional immune checkpoint receptors.

Herein, we aimed to develop and characterize a TIGIT-based CSR in the form of a chimeric receptor composed of TIGIT and CD28. We also describe an original in vitro model of T-cell hypofunction induction upon repetitive antigen exposure, in which this TIGIT CSR was able to enhance T-cell function. We were able to express high level of this chimeric receptor and we demonstrated its enhancing potential both in vitro*,* but more importantly, in a xenograft mouse model of human tumors.

## Methods

### Patient PBMCs and cell lines

All of the PBMCs used in this study were from healthy donors obtained from the Israeli Blood Bank (Sheba Medical Center, Tel-Hashomer, Israel). Melanoma cell lines HLA-A2+/MART-1+ (624.38) and HLA-A2^−^/MART-1^+^ (888) were generated at the Surgery Branch (National Cancer Institute, National Institutes of Health, Bethesda, MD) as described previously [30]. 888A2 is an HLA-A2-transduced line derived from 888. SK-MEL23 is a HLA-A2^+^ melanoma cell line (CVCL_6027). A375 (CVCL_0132) melanoma is HLA-A2^+/^MART-1^−^. Adherent cells were cultured in DMEM (Invitrogen, Carlsbad, CA), supplemented with 10% heat-inactivated Fetal Bovine Serum (Biological Industries, Beth Haemek, Israel) and were maintained in a 37 °C and 5% CO_2_ incubator. CD19-expressing B-cell targets were Raji (CCL86), JY (CVCL_0108), 721.221 (CVCL_6263), Nalm6 (CVCL_0092). K562 (CCL_243; which is CD19 negative) was engineered to express the CD19 antigen following retroviral transduction with a CD19 encoding vector. Non-adherent tumor cells were cultured in RPMI (Invitrogen, Carlsbad, CA), supplemented with 10% heat-inactivated Fetal Bovine Serum (Biological Industries, Beth Haemek, Israel) and were maintained in a 37 °C and 5% CO_2_ incubator. Lymphocytes were cultured in BioTarget medium (Biological Industries, Beth Haemek, Israel) supplemented with 10% heat-inactivated FBS and 300 IU/ml IL-2 (Peprotech, Israel) and maintained at 37 °C and 5% CO_2_.

### TCR and TIGIT chimeras retroviral constructs

**Fig. 1:**
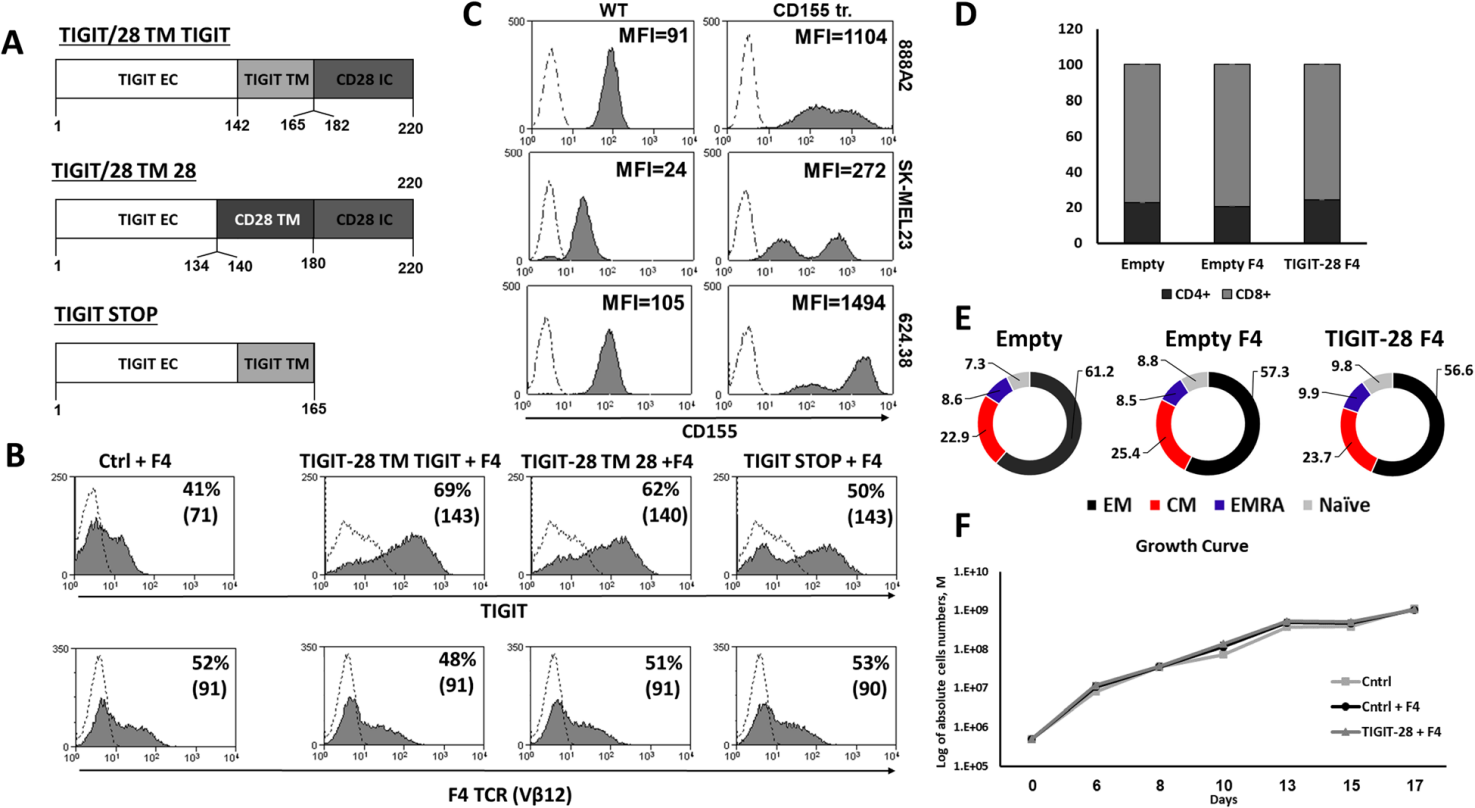
Design and expression of TIGIT-based CSRs, TCR F4 and CD155 ligand.**a** Schematic representation of the different TIGIT chimeras (as indicated). The amino acid numbering (based on the original protein) is indicated below each segment. **b** Human PBLs were transduced with the retroviral vectors encoding the indicated constructs. 72 h after transduction, the expression of the transgenes was measured by flow cytometry using antibodies specific for TIGIT (upper panels) and F4-TCR (Vβ12 – lower panels). The dotted line represents the basal endogenous expression in the control population. The percentage of positive cells and the MFI (in brackets) are shown. These results are representative of ten independent experiments with at least eight different donors and the difference between the population transduced and the non-transduced population was found statistically significant (*p* < 0.05; calculated using a *Student’s* paired t-test). **c** CD155 expression by melanoma lines (as indicated on the right side) was assessed by flow cytometry. The CD155 expression levels by native cell lines (left column – “WT”) and by CD155-transduced cell lines (right column – “CD155 tr.”) are shown. These results are representative of four independent experiments and the difference between the CD155-stained population and the control population (isotype-stained – dotted line) was found statistically significant (*p* < 0.05; calculated using a *Student’s* paired t-test). **d**-**f** Following transduction with TIGIT-28 or a control gene (tr.CD34), we measured the distribution of CD4+/CD8+ cells after a 10-day culture (**d**). No statistically significant difference was observed between the TIGIT-28 and control populations. These cells were stained also for CD45RO and CCR7 expression to determine the memory phenotype of these different populations (**e**). EM - Effector memory (CD45RO^+^/CCR7^−^), CM - central memory (CD45RO^+^/CCR7^+^), EMRA - terminally differentiated effector memory cells re-expressing CD45RA (CD45RO^−^/CCR7^−^) or naïve cell population (CD45RO^+^/CCR7^+^) are presented. No significant differences were observed in the distribution of these populations between the different treatments (i.e., TIGIT-28 or controls). These results are representative of three independent experiments with three different donors. **f** Cell count of these cells following transduction with TIGIT-28 + TCR F4, TCR F4 only or mock transduced was determined at different time points as indicated. No significant differences were observed and these results are representative of three independent experiments with three different donors

The α and β chains from the previously characterized TCRs specific for MART-1_26-35_ termed F4 (or DMF4) and F5 (or DMF5) were subcloned into the MSGV1 vector as described previously [30]. Similarly, we synthesized and cloned an anti-CD19-BBz CAR into this vector. The chimeras TIGIT-28 TM TIGIT (TMTi) and TIGIT-28 TM 28 (TM28) were created by overlapping PCR and their amino acid composition is indicated in Fig. 1a. A truncated version of TIGIT, TIGIT-STOP was produced by amplifying and cloning the TIGIT cDNA between 1 and 165 aa, followed by a stop-codon. The retroviral vector backbone used in this study, pMSGV1, is a derivative of the MSCV-based splice-gag vector (pMSGV), which uses a murine stem cell virus (MSCV) long terminal repeat and has been previously described [31].

### Transduction of PBLs

For transient virus production, transfection of 2.5 × 10^5^ 293GP cells with 2 μg DNA of MSGV1-based retroviral construct and 1 μg envelop plasmid (VSV-G) was performed using JetPrime transfection reagent (Polyplus, France). After 4 h, the medium was replaced. Retroviral supernatant was collected 48 h after the DNA transfection. Freshly isolated PBLs were stimulated in the presence of 50 ng/ml OKT3 (eBioscience, San Diego, CA). 2 days after stimulation, lymphocytes were transduced consecutively, first with a TCR or CAR, and 24 h after this, with supernatant encoding the CSR or control. Transduction was performed in non-treated tissue culture dishes (Nunc, Rochester NY) that had been pre-coated with RetroNectin (Takara, Japan) and retroviral vectors as previously described [30].

### Flow cytometry analysis and mAb

Fluorophore-labeled anti-human CD4, CD8, CD25, CD69, CD137, CD134 (OX40), TIGIT, CD155, CCR7, CD45RO and CD34 were purchased from BioLegend (San Diego, CA). Anti-Vβ12 antibody specific for F4 TCRβ was purchased from Beckman-Coulter/Immunotech (Marseille, France). Biotinylated Protein-L was purchased form Genscript (Piscata, NJ). Immunofluorescence, analyzed as the relative log fluorescence of gated live cells, was measured using a CyAn-ADP flow cytometer (Beckman Coulter, Brea). Approximately 1 × 10^4^ to 1 × 10^5^ cells (gated on live lymphocytes) were analyzed. Cells were stained in a FACS buffer made of PBS, 0.5% BSA, and 0.02% sodium azide.

### Cytokine release assays

PBL cultures were tested for reactivity in cytokine release assays using commercially available ELISA kits for IFNγ, IL-2 and TNFα (R&D Systems, Minneapolis, MN). For these assays, 1 × 10^5^ responder cells (PBL) and 1 × 10^5^ stimulator cells (tumor cells) were incubated in a 0.2-ml culture volume in individual wells of 96-well plates. Stimulator cells and responder cells were co-cultured for 18 h. Cytokine secretion was measured in culture supernatants diluted to be in the linear range of the assay.

### Cell separation

T-cell populations were separated using a magnetic beads-based approach for negative selection (EasySep TM - StemCell Technologies Inc., Canada).

### Intracellular staining

Following a 30-min co-culture of 7 × 10^5^ transduced T-cells with 3 × 10^5^ melanoma targets, the cells were fixed with formaldehyde 5% and permeabilized using ice-cold 90% methanol for 20 min. Then, the cells were washed in FACS buffer, stained for either phosphorylated ERK (clone D13.14.4E - Cell Signaling Technology, Danvers MA) or Bcl-xL (clone 7B2.5 – Southern Biotech, Birmingham AL) expression using a specific antibody and analyzed by flow cytometry, gated on the lymphocyte population.

### Cell mediated cytotoxicity assay

Target cells were co-cultured with transduced lymphocytes at 37 °C for 4 h, at E:T ratio of 1:3,1:6 and 1:12. All wells was completed to final volume 100ul. After the co-culture, equal volume of CytoTox-ONE™ (Promega, Madison, WI) according to manufacturer’s manual.

### In vitro hypofunction induction upon repetitive antigen exposure assay

1 × 10^6^ transduced lymphocytes were co-cultured with 1 × 10^5^ tumor target cells. Every 2 days, the effector cells were transferred to a new culture vessel in which 1 × 10^5^ tumor cells were previously seeded (Fig. 5a). This was repeated 4 times (i.e. a total of 8 days). At the end of this 8-day co-culture, these T-cells were tested in different assays as indicated.

### Established tumor assay

6–12 weeks year-old NOD/SCID/Gamma mice (Harlan, Jerusalem, Israel) were subcutaneous injected with 1 × 10^6^ SK-MEL23/155 cells resuspended in 100 μl HBSS medium (Biological Industries, Beth Haemek, Israel) and 100 μl Cultrex matrix (Trevigen). Two intravenous injections of 5 × 10^6^ transduced lymphocytes resuspended in 200 μl HBSS medium were performed at day 7 and 10 after tumor inoculation. Tumor size was measured every 2–3 days using a caliper in a blinded fashion. All the procedures were performed according to the guidelines of the university committee for animal welfare.

## Results

### Design and expression of TIGIT-chimeric constructs

The TIGIT receptor is a T-cell co-inhibitory molecule capable of downregulating T-cell function via binding to its ligands, often overexpressed by tumor cells. We aimed to take advantage of the presence of inhibitory ligands expressed by tumor cells to boost T-cell function using a costimulatory retargeting molecule. To this end, we designed and evaluated two TIGIT-based CSRs as described below. We hypothesized that such chimeric receptor could successfully convey positive signals to T cells following binding to TIGIT ligands. These TIGIT-based chimeras were constructed by fusing the extracellular domain of TIGIT to intracellular portion of the CD28 molecule (TIGIT-28) using a transmembrane (TM) portion derived from either TIGIT or CD28 (Fig. 1a). To enable the antigen-specific recognition of the tumor target cells, we utilized the MART1-specific TCR F4 previously characterized and used in clinical trials [32]. Following transduction of the chimeras and the TCR into primary human T-cells, we tested the expression of these molecules by flow cytometry. To negate any difference in function deriving from a differential TCR expression between the examined experimental groups, we first performed a TCR transduction step and then used these cells for subsequent transduction with the TIGIT or control construct. We also carefully and constantly controlled for equal TCR expression following transduction. As depicted in Fig.1b, we were able to express both TIGIT-28 constructs (TM TIGIT and TM CD28) in human T-cells efficiently without any selection. However, TM TIGIT (i.e. that contained the native TIGIT TM domain) was better expressed than TM CD28–69% (MFI = 143) vs. 62% (MFI = 140) of positive cells (*p* < 0.05). As aforementioned, the levels of expression of F4-TCR were similar between all tested groups (approximately 50%, with MFI = 90). Overall, these levels of expression by transduced PBLs cultured in vitro was sustained for more than 30 days without selection (data not shown).

TIGIT has been shown to bind to two ligands - CD155 and, with a lesser affinity, CD112. CD155 is a co-inhibitory ligand expressed on multiple human malignant tumors, including melanoma cells [15, 16, 18, 33]. Thus, we examined CD155 expression level on multiple melanoma lines. As seen in Fig. 1c, all the melanoma lines we tested expressed significant levels of CD155 (ranging from MFI = 24 to 105; *p* < 0.05). Additionally, to examine the impact of CD155 expression on T-cell function, 888A2, SK-MEL23 and 624.38 melanoma lines were transduced to enforce CD155 expression (Fig. 1c).

Following transduction with TIGIT-28 or a control gene (tr.CD34), we measured the distribution of CD4+/CD8+ cells after a 10-day culture. As seen in Fig. 1d, we did not observe a statistically significant difference between the TIGIT-28 and control populations with an approximate CD4/CD8 ratio of 20%/80%. Similarly, we also assessed the memory phenotype of these different populations by staining them for CD45RO and CCR7 expression and dividing them into effector memory, central memory, EMRA (terminally differentiated effector memory cells re-expressing CD45RA) or naïve cell population. No significant differences were observed in the distribution of these populations between the different treatments (i.e., TIGIT-28 or controls). Finally, we also followed the cell count of these cells following transduction with TIGIT-28 + TCR F4, TCR F4 only or mock transduced. As seen in Fig. 1f, we observed a similar cellular growth pattern between the different group. Overall, the transduction of TIGIT-28 did not significantly alter CD4/CD8 ratio, memory phenotype or in vitro growth pattern of engineered T-cells.

### TIGIT-28 (TM TIGIT) enhances T cell function and cytokine secretion

**Fig. 2:**
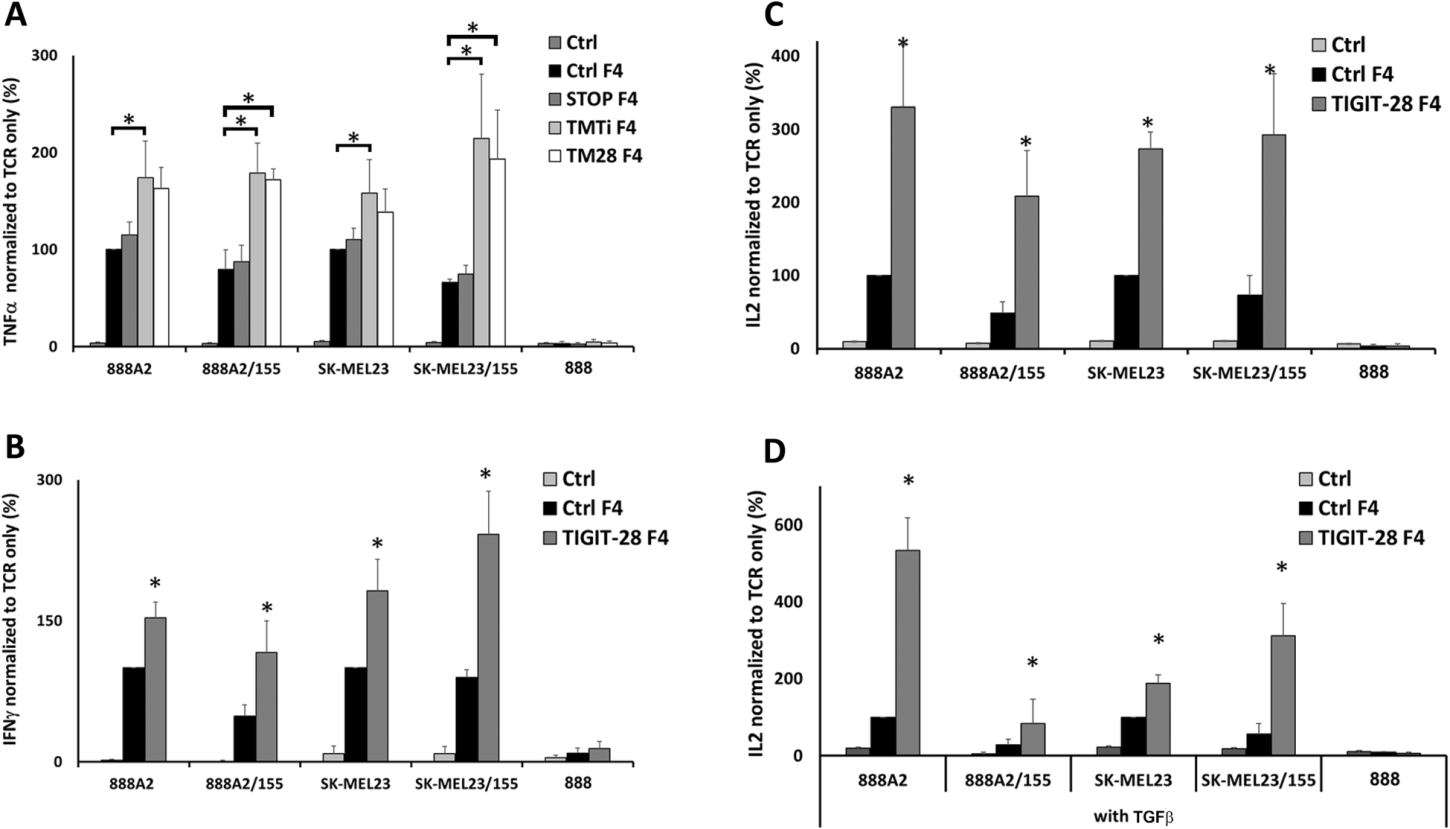
Anti-tumor activity of TIGIT-28 F4-transduced T cells. **a** Human PBLs expressing the F4 TCR were transduced with either TIGIT-28 (TMTi) or TIGIT-28 (TM28), TIGIT-STOP (truncated control) or mock (control). These cells were co-cultured with different melanoma line targets as indicated (x-axis). TNFα secreted in the co-culture supernatant was measured by ELISA. Cytokine concentrations were normalized for each target cell line (with or w/o CD155) to the secretion observed in the positive control Ctrl/F4 group in co-culture with the parental cell line (with an average TNF-α secretion of 4601 pg/ml for 888A2 and 3250 pg/ml for SK-MEL23). These results are presented as mean + SEM of three independent experiments, performed with three different donors (*: *p* < 0.05, calculated using a *Student’s* paired t-test). **b** Human PBLs expressing the F4 TCR were transduced with TIGIT-28 (TMTi) or mock transduced (Ctrl) and were co-cultured with different melanoma line targets as indicated. IFNγ secreted in the co-culture supernatant was measured by ELISA. As earlier, cytokine concentrations were normalized for each target cell line (with or w/o CD155) to the secretion observed in the positive control Ctrl/F4 group (with an average IFNγ secretion of 4620 pg/ml for 888A2 and 3350 pg/ml for SK-MEL23). These results are presented as mean + SEM of six independent experiments, performed with at least five different donors (*:*p* < 0.05, calculated using a *Student’s* paired t-test). **c**-**d** Similarly, engineered T-cells were co-cultured with different melanoma lines as indicated without TGFβ (**c**) or with 1.25 ng/ml of TGFβ (**d**). IL-2 secreted in the co-culture supernatant was measured by ELISA and its concentration was normalized for each target cell line (with or w/o CD155) to the secretion observed in the positive control Ctrl/F4 group (with an average IL-2 secretion of 488 pg/ml for 888A2 and 87 pg/ml for SK-MEL23 without TGFβ; and an average IL-2 secretion of 133 pg/ml for 888A2 and 67 pg/ml for SK-MEL23). These results are presented as mean + SEM of four independent experiments, performed with four different donors (*: *p* < 0.05, calculated using a *Student’s* paired t-test)

After establishing our experimental system, we tested the biological activity of both TIGIT-28 constructs (TM TIGIT and TM CD28) and their capacity to enhance a TCR-driven antigen specific response. Human primary T-cells transduced to express a TIGIT-28 chimera (TM TIGIT or TM28) along with the F4 TCR were co-cultured with several melanoma lines. Then, we assessed the secretion of cytokines important for anti-tumor T cells responses, namely TNFα, IFNγ and IL-2 [34]. As shown in Fig. 2a, TIGIT-28 chimeras significantly enhanced TCR F4 TNFα secretion when compared to control transduced cells or even to a truncated TIGIT receptor (TIGIT-STOP) that did not include the CD28 moiety. For example, when normalizing TNFα- secretions to that observed in the control TCR F4 only group (100% - equivalent to an average of 4601 pg/ml against the 888A2 target), we observed an average increase of 74% in the TM TIGIT group and of 62% for TM28, in co-cultures with 888A2 target cell line (*n* = 3; *p* < 0.05). Additionally, we observed only a 15% improvement in TNFα secretion in the TIGIT-STOP (control) group which clearly indicates that the CD28 portion is essential for the improved function of the CSR. Overall, as TIGIT-28 with the transmembrane portion of TIGIT (TIGIT-28 TM TIGIT) was better expressed and functioned superiorly compared to that with TM28, we selected it as the lead CSR for further assays. Henceforth, TIGIT-28 TM TIGIT will be termed TIGIT-28 in this study. As seen in Fig. 2b-c and similarly to TNF, we observed superior secretion of IFNg and IL-2 in T-cells expressing TIGIT-28 compared to the control TCR only group (e.g., up to 2.8 times more IFNγ in co-culture with the SK-MEL23 cells line – Fig. 2b). Notably, no-significant cytokine secretion was measured in control co-cultures with HLA-A2^−^ 888 melanoma cells.

T-cells encounter a hostile environment when interacting with solid tumors. One of the main immunosuppressive protagonists is the cytokine TGFβ which can dampen critically T-cell function, T cell proliferation and IL-2 production [35, 36]. Since TIGIT-28 mediated an increase in cytokine secretion in the presence of CD155 (Fig. 2a-c), we sought to examine if this pattern would be preserved in the context of an additional inhibitory stimulus. We set up an overnight co-culture with melanoma cells in the presence of soluble TGFβ (1.25 ng/ml) and measured IL-2 secretion in the supernatant by ELISA. As anticipated, IL-2 secretion was reduced in the presence of TGFβ, when the target did or did not overexpress CD155 (Fig. 2d). Nevertheless, TIGIT-28 transduced T-cells maintained a higher secretion compared to the control (TCR only) in the presence of TGFβ (an average of 708 pg/ml vs. 137 pg/ml in co-culture with 888A2; *p* < 0.05). This suggests that TIGIT-28 transduced cells may function better in a hostile tumor microenvironment compared to unmanipulated cells. In conclusion, TIGIT-28-expressing T cells demonstrate an improved anti-tumor cytokine secretion capability.

### Activation marker upregulation and increased pERK and Bcl-xL levels in TIGIT-28 transduced T-lymphocytes

**Fig. 3:**
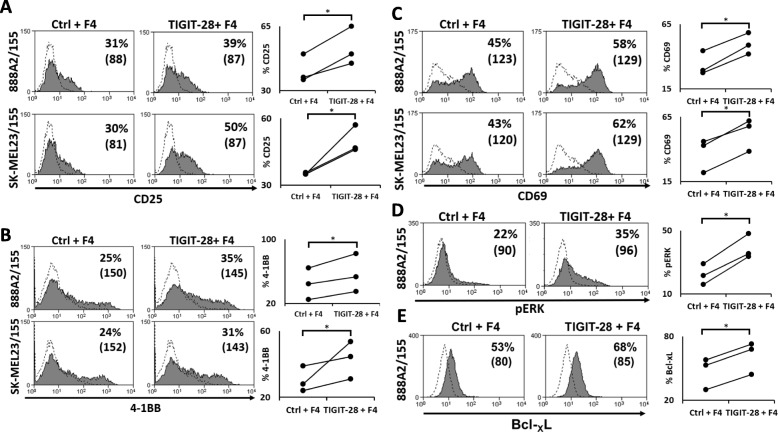
Activation marker upregulation and increased phosphorylation of ERK protein by TIGIT-28 transduced T-lymphocytes. **a**-**c** Transduced PBLs with either TIGIT-28-F4 or Ctrl-F4 cells were co-cultured with melanoma lines (as indicated on the left side) and analyzed by flow cytometry for activation marker expression (CD25 (**a**), 4-1BB/CD137 (**b**) and CD69 (**c**)) gated on the CD8^+^ population (as indicated). The percentage of positive cells and the MFI (in brackets) are shown. These results are representative of at least three independent experiments (summary results shown in the right panels) with at least three donors and the difference between TIGIT-28 and the control was found to be statistically significant (*:*p* < 0.05, calculated using a *Student’s* paired t-test). **d**-**e** Transduced PBLs with either TIGIT-28-F4 or TCR F4 only (control) cells were incubated with 888A2/155 melanoma line for 30 min. (for pERK- in 5D) or overnight (for Bcl-xL – in 5E) and analyzed for intracellular levels of these proteins. These results are representative of three independent experiments (summary results shown in the right panels) and the difference between the two groups was found statistically significant (*p* < 0.04, calculated using a *Student’s* paired t-test)

TIGIT can directly inhibit T cell proliferation and lower their activation phenotype including the downregulation of CD69 and CD25 markers [9]. Thus, we sought to determine if TIGIT-28 could counteract this and enhance the expression of T-cell activation markers such as CD25, CD69 and 41BB (CD137). To this end, the expression of these activation markers was assessed on TIGIT-28/F4 or F4 (control) transduced T-cells that were co-cultured with different targets. Compared to the control T-cell population, TIGIT-28-engineered cells demonstrated a statistically significant enhanced expression of these markers: for instance, for CD25, we detected 50% of positive cells for TIGIT-28 vs. 30% for the control (Fig. 3a; *p* < 0.05). Similarly, we noted a proportion of 31% of positive cells for 41BB in the TIGIT-28 sample compared to 24% in the control one (Fig. 3b; *p* < 0.05) and of 58% vs. 45% for CD69 respectively (Fig. 3c; *p* < 0.05).

Activation of the CD28 pathway has been shown to enhance the activation and survival of T-cells via several signaling molecules such as pERK and Bcl-xL [37, 38]. We thus assessed if TIGIT-28 engineered T-cell may actively augment ERK phosphorylation in co-cultures with tumor cells. TIGIT-28- or control-transduced TCR F4 T-cells were incubated with target melanoma cells for 30 min and then analyzed for intracellular pERK expression. As seen in Fig. 3d, we observed a significant increase in pERK expression (e.g. 35% vs. 22% of pERK-positive cells respectively; *p* < 0.05). No significant pERK elevation was observed in control co-cultures with the melanoma line 888 (not shown). We also examined whether TIGIT-28 could lead to an increased Bcl-xL expression in F4 transduced T cells. Following an overnight co-culture, we performed an intracellular staining of engineered T-cells with anti-Bcl-xL. As depicted in Fig. 3e, Bcl-xL expression in TIGIT-28/F4 was upregulated compared to that assessed in F4 control cells (29% vs 3%; *p* < 0.05). These results demonstrate that TIGIT-28 is capable of activating the CD28 signaling cascade.

### TIGIT-28 can enhance the function of high affinity TCR expressing T-cells

**Fig. 4:**
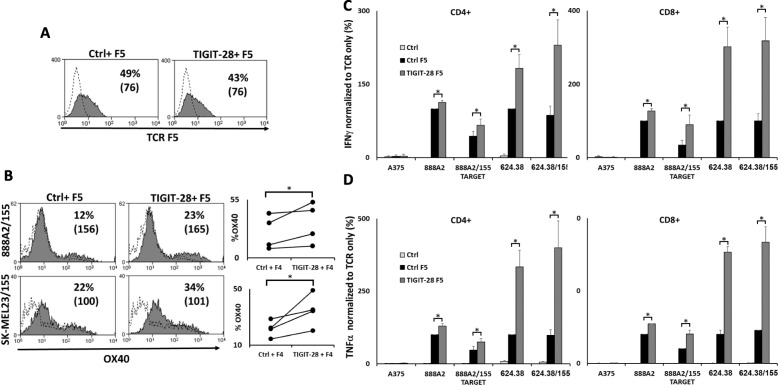
TIGIT-28 enhances the function of high affinity TCR F5. **a** Primary human T-cells engineered to express the MART-1-specific high-affinity TCR F5. These cells were co-transduced with TIGIT-28 or a control gene (tr. CD34) were analyzed for TCR expression using a MART-1/HLA-A02 tetramer by flow cytometry. The dotted line represents the basal endogenous expression in the control population. The percentage of positive cells and the MFI (in brackets) are shown. **b** These cells were co-cultured with the indicated targets and analyzed for OX40 expression 24 h after the beginning of the co-culture. The percentage of OX40 positive cells (gated on the CD4+ population) and the MFI (in brackets) are shown in the left panels. These results are representative of four independent experiments (indicated in the right panel plots) with at least three donors and the difference between TIGIT-28 and control vector was found to be statistically significant (*p* < 0.05, calculated using a Student’s paired t-test). **c**-**d** These engineered T cells were separated into either CD4^+^ or CD8^+^ populations using magnetic beads. Separated cells were co-cultured with the indicated targets. The concentrations of TNFα (**c**) and IFNγ (**d**) secreted in the co-culture supernatant were detected by ELISA. Cytokine concentrations were normalized for each target cell line (with or w/o CD155) to the secretion observed in the positive control F5-TCR only group (for CD4^+^ cells - left panels: normalized to an average TNFα secretion of 19,863 pg/ml for 888A2 and 1802 pg/ml for 624.38. and to an average IFNγ secretion of 13,997 pg/ml for and 3876 pg/ml for 624.38; for CD8^+^ cells - right panels: normalized to an average TNFα secretion of 25,478 pg/ml for 888A2 and 3867 pg/ml for 624.38. and to an average IFNγ secretion of 21,249 pg/ml for 888A2 and 5696 pg/ml for 624.38). These results are averages of at least 5 independent experiments, performed with at least 3 different donors (*:*p* < 0.05, calculated using a *Student’s* paired t-test)

The affinity range of TCRs to their cognate MHC/peptide complex can differ in 1–2 orders of magnitude between different receptors targeting the same complex [39, 40]. To assess the potential benefit of TIGIT-28 in the context of a high-affinity TCR, we made use of the MART1 specific TCR DMF5 (F5) and investigated whether TIGIT-28 could also enhance its function. We previously showed that as a CD8-independent TCR, the F5 TCR can also function in CD4^+^ T cells [30]. To test the potential beneficial effect of TIGIT-28 in CD4^+^ T-cells, we co-cultured T-cells transduced with either Ctrl/F5 or TIGIT-28/F5 (Fig. 4a) with several targets and stained these cells for OX40 (CD134) - a classical CD4^+^ activation marker expression [41]. We noted a proportion of 23% of OX40 positive cells in the TIGIT-28 sample compared to 12% in the control one (Fig. 4b). TIGIT-28/F5 transduced T-cells were then separated into CD4+ and CD8+ population using magnetic beads. The cells were co-cultured with different human melanoma lines and following this, we measured secretion of TNFα and IFNγ (Fig. 4c-d); when normalized to the activity of F5 TCR only T-cells against different targets, both CD4^+^ and CD8^+^ T-cells transduced to express TIGIT-28/F5 secreted higher levels of cytokines than the control TCR-only transduced T cells (e.g. approximately 20–60% more IFNγ and TNFα secretion were observed in co-culture with different targets expressing high levels of CD155; *p* < 0.05). Thus, TIGIT-28 can generically improve the function of CD4^+^ and CD8^+^ T-cells expressing a high-affinity TCR.

### TIGIT-28 can enhance the function of CAR T-cells depending on CD155 expression

**Fig. 5:**
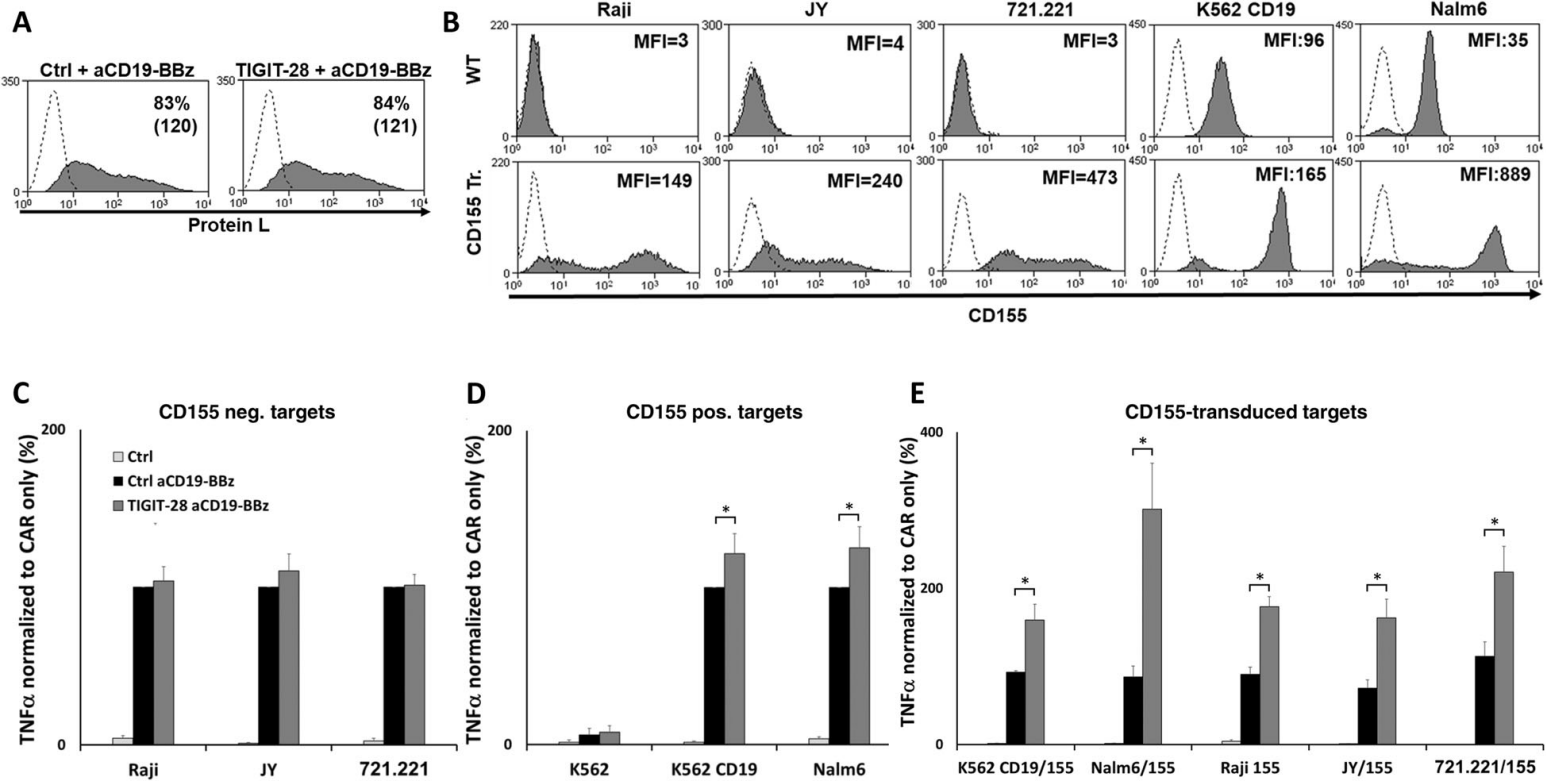
TIGIT-28 enhances the function of anti-CD19-BBz CAR-T cells. **a** primary T-cells engineered to express the a CD19-specific CAR were co-transduced with TIGIT-28 or a control gene (tr. CD34) and analyzed for CAR expression by flow cytometry using protein-L staining. **b** Different target cells were transduced with a retroviral vector encoding CD155. CD155 expression in the native (WT) or the CD155-engineered (CD155 tr.) target cell lines was assessed by flow cytometry. The percentage of positive cells (indicated by the grey surface) and the MFI (in brackets) are shown. These results are representative of 6 independent experiments. **c**-**e** Human T-cells were engineered to express a 2nd generation (41BB-based) CD19-specific CAR and co-transduced with TIGIT-28 or a control gene (tr. CD34). These cells were co-cultured with CD155 negative target cells (**c**), native CD155 positive target cells (**d**), and CD155-transduced target cells (**e**). TNFα secreted in the co-culture supernatant was measured by ELISA. Cytokine concentrations were normalized for each target cell line (with or w/o CD155) to the secretion observed in the positive control Ctrl/CD19-BBz group with an average TNFα secretion of 5884 pg/ml for Raji, 4558 pg/ml for JY, 4330 pg/ml for 721.221, 8102 pg/ml for K562-CD19 and 3902 pg/ml for Nalm6. These results represent the mean + SEM of at least 6 independent experiments, performed with 6 different donors (*: *p* < 0.05, calculated using a *Student’s* paired t-test)

In addition to classical TCRs, we also sought to examine if TIGIT-CD28 could improve the function of another type of activating receptor such as chimeric antigen receptor (CAR). We chose to focus on a CD19 specific 2nd generation CAR that incorporate the 41BB signaling moiety. T-cells transduced to express both CAR and TIGIT-28 CSR (or mock-control) (Fig. 5a). In parallel, we also sought to determine to what extent TIGIT-28 functional enhancement was dependent on CD155 expression by targets cells. To this end, we assessed CD155 expression on different CD19^+^ target cells. As seen in Fig. 5b, we could not detect any CD155 surface expression on Raji, JY and 721.221 targets while K562/CD19 and Nalm6 expressed considerable levels. In parallel, these tumor lines were retrovirally transduced with a construct encoding CD155 and the expression of CD155 by these engineered target cells is also shown in Fig. 5b (lower panels). We then co-cultured these native or CD155 engineered CD19-expressing targets (or antigen negative K562 control) with CD19-CAR T-cells also transduced to express TIGIT-28 (or a control gene). As seen in Fig. 5c-e, TIGIT-28 was able to mediate an increased secretion of cytokine compared to control (up to 50% more TNFα in co-culture with K562-CD19/155; *p* < 0.05). Interestingly, no significant enhancement by TIGIT-28 was noted in co-cultures with CD155-negative targets (Fig. 5c) indicating that TIGIT-28 function is dependent on CD155 expression by the target cells. To ascertain this, we also plotted relative improvement in TNFα secretion by the TIGIT-28 population over that of the control population as a function of CD155 expression by the target cells and we observed a significant correlation (R^2^ = 0.8923 using linear regression; Additional file 1: Figure S1) between both parameters.

In conclusion, TIGIT-28 can improve CAR-T cell function and this enhancement is dependent on CD155 expression by target cells.

### TIGIT-28 can help rescuing hypofunctional T cells

**Fig. 6:**
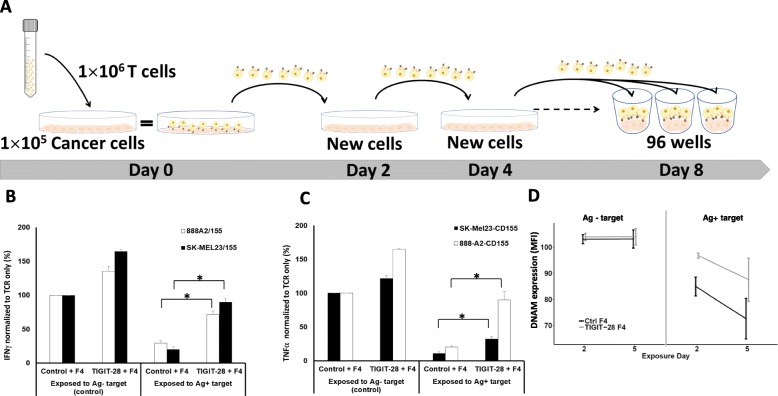
TIGIT-28 can help mitigating T cell hypofunction. **a** Schematic representation of the hypofunction induction assay following repetitive antigen exposure developed herein. Initially, 10^5^ cancer cells were seeded in a 6-well plate and 4 h later 10^6^ transduced T cells were added (day = 0). 2 days afterwards, previously co-cultured T-cells were transferred to a new plate, previously seeded with 10^5^ new cancer cells. This process was repeated 4 times, totaling 8 days of co-culture. On day 8, these T-cells were used in an additional co-culture for cytokine secretion assessment. **b**-**c** TIGIT-28/F4 or ctrl/F4 transduced T cells were conditioned in co-culture with 888A2 (antigen positive) or HeLa (antigen negative - control) for 8 days as described above. On day 9, these T cells were co-cultured with either 888A2/155 or SK-MEL23/155. **b** IFNγ or **c** TNF-α secreted in the co-culture supernatant was measured by ELISA. Cytokine concentrations were normalized to the secretion observed in the positive control Ctrl/F4 group exposed to antigen negative tumor cells (HeLa) for 8 days (with an average secretion of IFNγ of 2530 pg/ml for 888A2, 1903 pg/ml for SK-MEL23 and with an average secretion of TNF-α of 2475 pg/ml for 888A2, 1170 pg/ml for SK-MEL23). These results are representative of three independent experiments, performed with at least two different donors (*: *p* < 0.05, calculated using a *Student’s* paired t-test). **d** The surface expression of CD226 (DNAM), an antagonist to TIGIT, was analyzed on TIGIT-28/F4 or Ctrl/F4 transduced T cells that were co-cultured with 888A2/155 (Ag^+^ target) or HeLa (Ag^−^ target) cells for several days. On day 2 and 5 (after the beginning of the conditioning co-culture), these cells were stained with anti-CD226 and analyzed by flow cytometry. These results are presented as the MFI average of three independent experiments, performed with at least two different donors (*: *p* < 0.05, calculated using a *Student’s* paired t-test)

Exhaustion/hypofunction of T cells following repetitive stimulation, a lack of positive co-stimulation and constant exposure to the immunosuppressive TME can greatly impair their anti-tumor function. Thus, we sought to examine if the concomitant expression of a CSR in TCR-transduced T-cells could rescue them from a hyporesponsive state [42] acquired over time following repetitive antigen exposure. To this end, we developed an in vitro experimental system to examine the function of exhausted T-cells; in this system, we performed long co-cultures of F4 only or F4 + TIGIT-28-transduced T cells with tumor cells which were replenished every 48 h for a duration of 8 days. As an additional control, T-cells were incubated during 8 days with an antigen-negative tumor and we surmised that in these conditions, T-cells would not reach an hypofunctional state. Then, these “exhausted/tumor challenged” T-cells were isolated and taken to a final co-culture experiment to assess their basic functionality against antigen-positive targets by means of cytokine secretion (see Fig. 6a).

As seen in Fig. 6b-c, after a period of 8 days, F4 only T-cells, which were subjected to long co-cultures with a MART1+/HLA-A2+ target cell line (888A2), displayed a dramatic reduction in cytokines secretion, akin to a hyporesponsive state; for example, when normalized to the IFNγ secretion observed by TCR-F4 only T-cells previously incubated with antigen negative targets during 8 days, that of T-cells incubated with 888A2 went down by 80% (i.e., 100% vs. 20.3% respectively) in co-cultures with the SK-MEL23/155 target. In contrast, TIGIT-28/F4 T cells exhibited much higher cytokine secretion profile (reaching in average 90.1% of the positive control; *p* < 0.05 – Fig. 6b). This beneficial effect mediated by TIGIT-28 was not due to a differential TCR expression following these long co-cultures as F4 TCR levels (measured by flow cytometry) were similar in T-cells exposed to antigen-negative, antigen-positive targets (Additional file 1: Figure S2). This demonstrates that TIGIT-28 is able to mitigate the effects of prolonged antigen exposure on T-cell function.

TIGIT can limit lymphocyte functionality by downregulating the expression of surface receptors such as CD226 (known also as DNAM1) which conveys positive signals [8]. This mechanism has further ramifications as TIGIT and DNAM1 directly compete for the binding of the ligand CD155 [14]. Thus, we also assessed DNAM1 expression in T cells subjected to this 8-day co-culture compared to that observed in T-cells before this treatment. As seen in Fig. 6d, TIGIT-28/F4 equipped cells were able to maintain, after several days in co-cultures with target cells, higher levels of DNAM1 surface expression compared to TCR F4-only T cells (87% vs. 72% of DNAM1 positive cells respectively on day 5 – Fig. 6d). Thus, TIGIT-28 may improve T-cell function and activation phenotype also in the case of continued challenge with tumor cells.

### TIGIT-28 mediates superior anti-tumor cytotoxicity in xenograft model

**Fig. 7:**
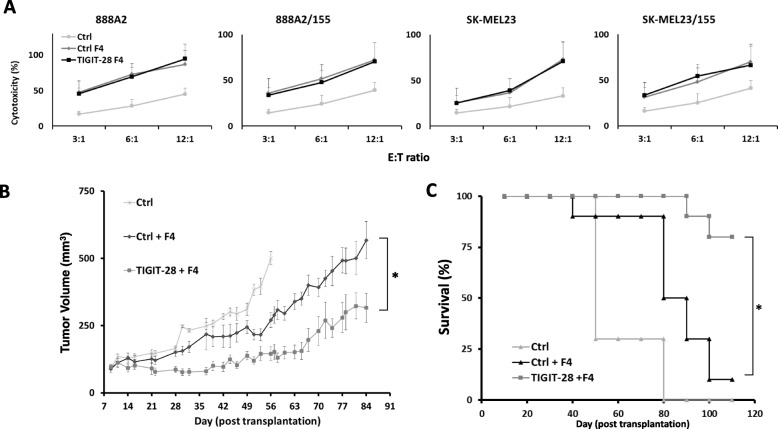
TIGIT-28 mediates superior anti-tumor cytotoxicity in xenograft models. **a** TIGIT-28/F4 or Ctrl/F4 transduced T-cells were co-cultured with the indicated target cell lines for 4 h at different E:T ratios (as indicated). Release of lactate dehydrogenase as a measure of cytotoxicity was analyzed as described in the Material and Methods section and normalized to that of target cells incubated with Triton X 100(9%). These results are representative of three independent experiments with three different donors and no significant different was noted between the TCR only group (Ctrl F4) and F4 + TIGIT28. **b**-**c** NSG mice inoculated with SK-MEL23/155 tumor cells and treated with TIGIT-28/F4, F4 TCR only (Ctrl/F4) T-cells or mock transduced T-cells (Ctrl.). *(B)* Tumor growth was measured in a blinded fashion using a caliper and calculated using the following formula: (Dxd^2^)xΠ/6, where D is the largest tumor diameter and d its perpendicular one. Results are shown for the different time points as mean + SEM (*n* = 10) and the difference between the TIGIT-28 and Ctrl-treated groups was found statistically significant (ANOVA; *p* = 4.2e-05). *(C)* The percentage survival per treated group was determined on a daily basis and is represented by Kaplan-Meier survival curve. The difference between the TIGIT-28 + F4 and F4 only-treated groups was found statistically significant (Logrank test; *p* = 0.0006)

To measure cytotoxicity exhibited by TIGIT-28 engineered T-cells, TIGIT-28/F4 or ctrl/F4 T cells were co-cultured with different targets for 4 h at different E:T ratios. As seen in Fig. 7a, we did not observe a significant difference between the two groups suggesting that TIGIT-28 expression did not impair cell-mediated cytotoxicity. Nevertheless, we assumed that a conventional 4-h cytotoxicity assay may not necessarily fully illustrate the anti-tumor activity of TIGIT-28 transduced T cells. We therefore decided to assess the anti-tumor function of TIGIT-28-transduced T-cells in vivo and examined the ability of these cells to suppress tumor growth in a human tumor xenograft mouse model. 1 × 10^6^ tumor cells (SK-MEL23/155) were injected in the flank of immunodeficient mice. One week afterwards, 5 × 10^6^ T cells (TIGIT-28/F4 or Ctrl/F4) were injected IV through the tail vein. We followed tumor growth and could demonstrate that TIGIT-28/F4 T-cells mediated a significant delay in tumor growth compared to the control group that was treated with control-F4 transduced T-cells (Fig. 7b; *n* = 10, *p* = 4.2e-5, measured by ANOVA). In two additional experiments, we also obtained a statistically significant difference between the TIGIT-28 and Ctrl-treated groups (with *n* = 5; *p* = 0.0003 and *n* = 5; *p* = 0.0018 – not shown). Moreover, at the endpoint, 83% of the TIGIT-28 treated mice survived compared to 16% in the control group (Fig. 7c). In conclusion, TIGIT-28-expressing T-cells could delay tumor growth and prolong significantly the survival of tumor-bearing mice.

## Discussion

The TIGIT/CD155 inhibitory axis is an attractive target as CD155 (PVR, necl-5) is overexpressed in multiple cancer types including colon cancer, lung adenocarcinoma, melanoma, ovarian, breast, pancreatic cancer and glioblastoma and its expression is correlated with poor prognosis and tumor proliferation [13, 17]. To derive benefit from inhibitory ligand overexpression on tumors, we designed and expressed a CSR in the form of TIGIT exodomain fused to CD28 endo-domain. While most of the CD28-based human CSRs incorporate a TM domain derived from CD28 [28, 43–46], we noticed in the present case that the TM of the original exodomain, i.e. TIGIT was more advantageous than that of CD28 (Figs. 1 and 2). This observation strengthens the need to evaluate CSR design empirically similarly to CARs [47]. Though we describe a CD28-based CSR prototype in this work, it is conceivable that the use of alternative/additional costimulatory endodomain (such as 41BB. ICOS, CD27 or OX40) to design 1st or 2nd generation CSR with poly-functionality, especially as we have showed in the past that additional co-stimulation is beneficial in the context of TCR gene transfer [25]. Moreover, since simultaneous blockade of TIGIT and PD-1 could enhance cytokine production, proliferation and degranulation in CD8^+^ TILs from melanoma patients, one could surmise that the combination of multiple CSRs based on TIGIT and PD1, each with different signaling moieties may act synergistically [19]. As relative levels of ligands, nature and affinity of the interaction, expression levels of the different chimeras can further influence T-cell function, it is nevertheless difficult to directly compare the function of PD1–28 and TIGIT-28 CSRs as they bind to different ligands (PDL1 and CD155 respectively) on the target cells. Conversely, TIGIT has been showed to bind to other ligands besides CD155, such as CD112 or CD113 (with lower affinity though) and thus, we might infer that TIGIT/28 may also be biologically functional when binding to these alternative ligands. Additionally, as TIGIT is also naturally expressed by NK cells, one may envisage that TIGIT-based CSR may improve their function, using either CD28 or perhaps a different signaling moiety such as from the DAP family [48].

T-cell antigen specificity may be redirected using either TCRs or CARs [24]. While one of the advantages of CARs is the incorporation a co-stimulatory moiety, these receptors are limited to membrane antigen targets. Alternatively, TCR can target also intracellular antigen but lack built-in co-stimulatory domains [49]. Thus, a major advantage of CSR lies with the possibility to selectively combine costimulatory signals in the context of TCR stimulation. Interestingly, we show that CSRs can be beneficial when being engaged concomitantly not only with medium-affinity but also with high-affinity TCR (e.g., using the F5 TCR, Fig. 4), though with the latter, we observed a lower enhancement of cytokine secretion (we measured an average enhancement in cytokine secretion of 162% ± 27% in co-cultures with T-cells expressing F4 TCR compared to 48% ± 13% with F5 TCR; *p* = 0.0042, compared using a paired *Student’s* t-test). Of importance, this superior function was also independent of the type of subpopulation both in CD8^+^ and CD4^+^ T cell populations (Fig. 4c-d) or of the type of antigen-targeting receptor employed; indeed, we also showed that the function of aCD19-BBz CAR could be enhanced when co-expressing TIGIT-28 (Fig. 5). In sum, these results underline the versatility of the CSR approach.

The ubiquitous nature of CD155 expression on different tumors makes TIGIT-28 a valuable switch receptor to be used in conjunction with receptors targeting tumor antigens widely expressed such as NYESO or p53 [24, 50]. Also, it was shown that TIGIT is upregulated by T-cells in chronic viral infections [13, 14]. Thus, it is possible that anti-viral strategies based on engineered T-cells may derive benefit from the use of TIGIT-28.

An additional advantage to the present strategy, when compared to antibodies/checkpoint inhibitors, lies with the permanent nature of this modification and with the fact that it provides engineered T-cells with the opportunity to activate costimulatory pathways; the latter could facilitate T-cell persistence over time, proliferation, differentiation into memory cells and improved performance in patients. It is also reasonable to conjecture that tumors may escape from an immune response and be selected over *time* in vivo based on their high levels of inhibitory ligands (in our case CD155) [12, 15, 17, 24]. However, when using CSRs, overexpression of inhibitory ligands by tumors may actually be detrimental to the latter and thus, this could alternatively lead to a decrease in immunosuppression and ultimately, to a broader T-cell anti-tumor response.

## Conclusion

In conclusion, we have demonstrated the efficacy of signal conversion by a novel CSR, namely the TIGIT-28 chimeric receptor that can work in concert with a TCR or CAR. TIGIT-28 can improve the anti-tumor activity of engineered T-cells in an antigen dependent setting leading to tumor regression. We are confident that this approach geared at the manipulation of costimulatory pathways bears important implications for the enhancement of T cell-based treatments using gene-transfer approaches.

## Additional files

Additional file 1: **Figure S1.** Correlation between ligand expression and chimera function. **Figure S2.** TCR expression levels following hypofunction induction. (PPTX 76 kb)

## Data Availability

n/a
