# Supplementary material for: A TIGIT-based chimeric co-stimulatory switch receptor improves T-cell anti-tumor function
Source: J Immunother Cancer. 2019 Sep 9;7:243. doi: 10.1186/s40425-019-0721-y (PMC6734436; doi:10.1186/s40425-019-0721-y)
Supplement: Supplementary file 1 — Figure S1. Correlation between ligand expression and chimera function. Figure S2. TCR expression levels following hypofunction induction. (PPTX 76 kb) [file 40425_2019_721_MOESM1_ESM.pptx]

## Slide 1
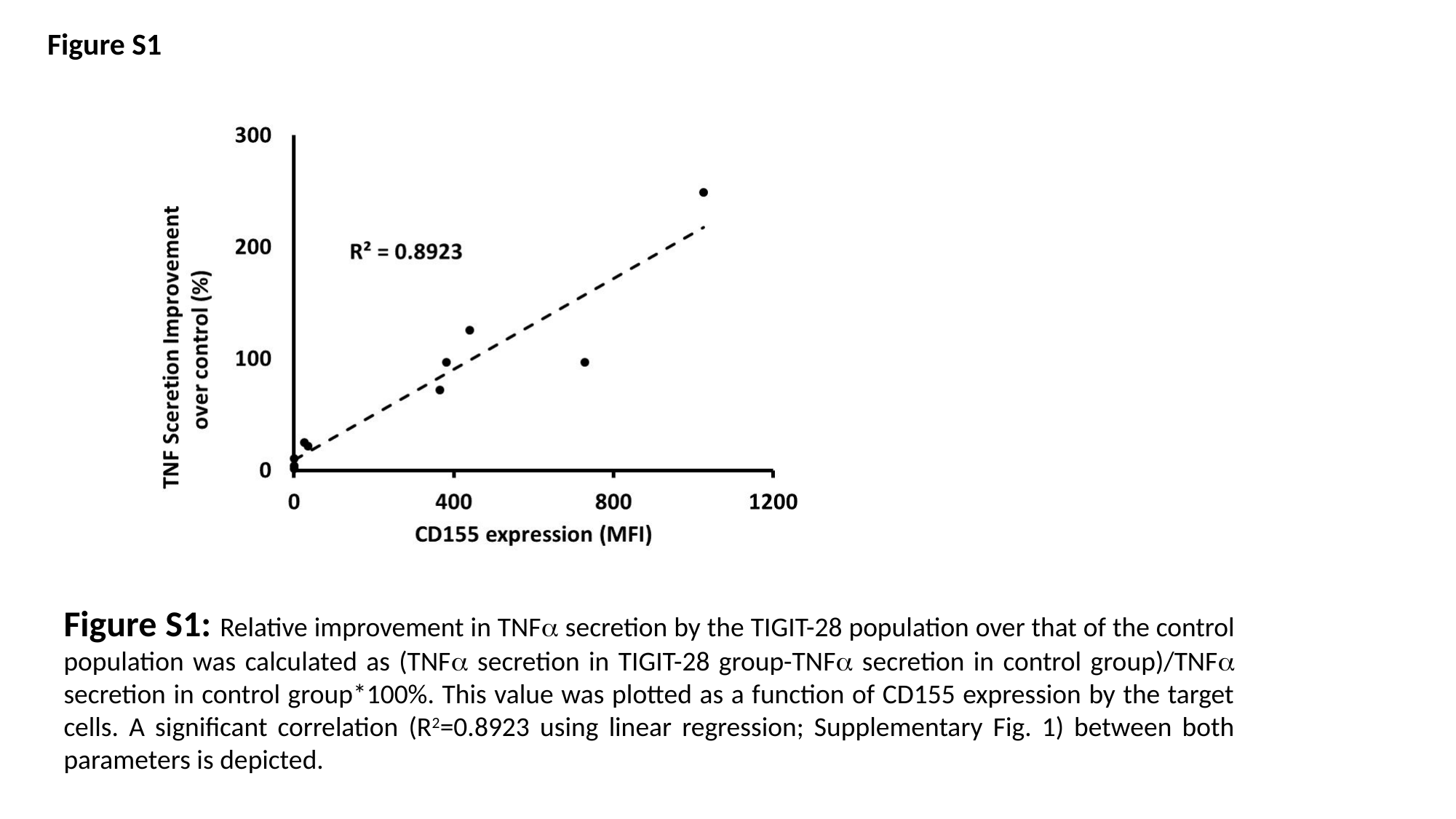

Figure S1
Figure S1: Relative improvement in TNFa secretion by the TIGIT-28 population over that of the control population was calculated as (TNFa secretion in TIGIT-28 group-TNFa secretion in control group)/TNFa secretion in control group*100%. This value was plotted as a function of CD155 expression by the target cells. A significant correlation (R2=0.8923 using linear regression; Supplementary Fig. 1) between both parameters is depicted.

## Slide 2
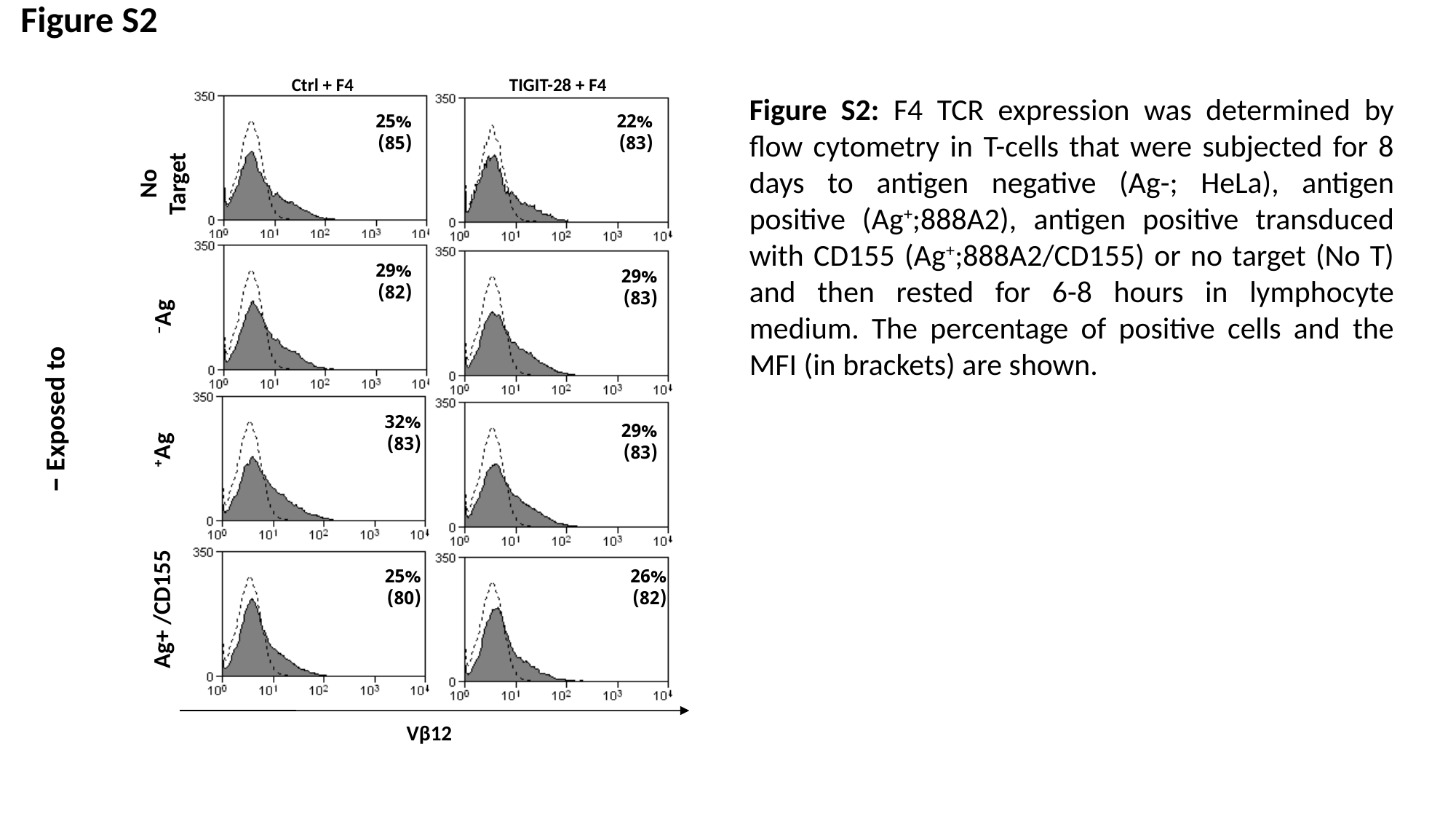

Figure S2
Ctrl + F4
TIGIT-28 + F4
Figure S2: F4 TCR expression was determined by flow cytometry in T-cells that were subjected for 8 days to antigen negative (Ag-; HeLa), antigen positive (Ag+;888A2), antigen positive transduced with CD155 (Ag+;888A2/CD155) or no target (No T) and then rested for 6-8 hours in lymphocyte medium. The percentage of positive cells and the MFI (in brackets) are shown.
25%
(85)
22%
(83)
No Target
29%
(82)
29%
(83)
Ag–
Exposed to –
32%
(83)
29%
(83)
Ag+
25%
(80)
26%
(82)
Ag+ /CD155
Vβ12
